# *S. gallolyticus* Aortic Valve Endocarditis with Mitral Valve Leaflet Aneurysm

**DOI:** 10.1155/2022/3111032

**Published:** 2022-12-16

**Authors:** S. Poddi, I. Tropea, G. Faggian, S. De Feo, M. Zordan, A. Rungatscher

**Affiliations:** ^1^Division of Cardiac Surgery, University of Verona Medical Center, Verona, Italy; ^2^Division of Cardiovascular diseases, Pederzoli Hospital, Peschiera del Garda, Italy; ^3^Division of Pathology, University of Verona Medical Center, Verona, Italy

## Abstract

*S. gallolyticus* is one of the pathogenic agents of endocarditis, and mitral valve aneurysm is a rare but potentially devastating complication. We present a case of *S. gallolyticus* aortic valve endocarditis with concomitant anterior mitral valve leaflet aneurysm. Patient underwent surgery before aneurysm perforation, and postoperative course was uneventful. Time of surgery is crucial to avoid severe complications due to aneurysm rupture.

## 1. Introduction

*Streptococcus gallolyticus* is one of the etiologic factors of infective endocarditis (IE); it is also associated with colonic neoplasia. Rarely, aortic valve (AV) infection may lead to a specific scenario involving mitral valve (MV) and causing MV anterior leaflet aneurysm. Infection can spread to MV through direct contact between aortic vegetation and MV leaflet, contiguous infection through mitral-aortic fibrosa, and/or regurgitant infectious flow from aortic valve [1, 2]. MV aneurysm is rare but mostly associated with IE; its acute rupture may cause massive mitral regurgitation and subsequent pulmonary edema and/or systemic embolization [3]. According to the current guidelines, indications for surgery in case of IE are uncontrolled infection, abscess or fistula formation, pseudoaneurysm, and large vegetation with embolic potential [4]. In case of MV aneurysm, despite no specific indication in the guidelines, prompt surgery may be indicated to avoid the aforementioned catastrophic complications. To the best of our knowledge, only few cases of endocarditis-related MV aneurysm are described; most of them have been treated by urgent surgery due to aneurysm rupture complications [1, 3, 5–14]. Herein, we present a case of a 78-year-old male with native aortic valve endocarditis with positive blood cultures for *S. gallolyticus* and intraoperative evidence of anterior MV leaflet aneurysm.

## 2. Case Presentation

**Figure 1:**
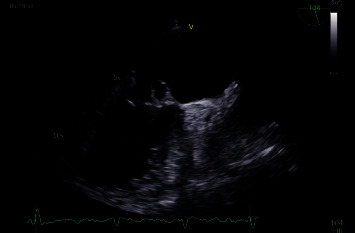
Echocardiogram shows anterior mitral valve leaflet aneurysm.

**Figure 2:**
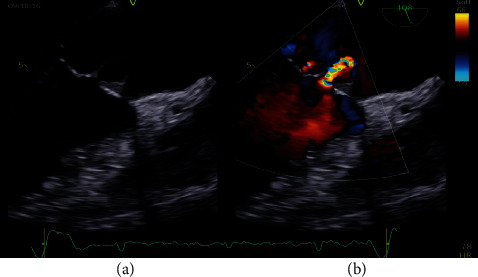
(a) MV aneurysm. (b) Aneurysm-related MV regurgitation.

IRB approval, consent statement, and clinical trial registration are not applicable for the present study. In January 2022, a 78-year-old male was referred to our center. His past medical history included diabetes mellitus, paroxysmal atrial fibrillation (AF), moderate aortic regurgitation, and moderate ascending aorta dilation. In November 2021, patient experienced dyspnea, high-rate AF, and low-grade fever; he had been admitted in a regional hospital and diagnosis was acute congestive heart failure (HF). Transesophageal echocardiogram (TEE) revealed a tricuspid aortic valve with mild regurgitation (2 + /4+; vena contracta 4 mm) and thin fibrotic striae on the arterial side of the cusps. It also revealed an isoechogenic vegetation-like formation (10 mm) on the atrial side of A2 scallop of the MV, associated with trivial-mild MV regurgitation (1 + −2+/4+). Left ventricular ejection fraction (EF) was preserved. Blood cultures were positive for *S. gallolyticus*. Treatment with ceftriaxone was started, and imaging found no sign of embolization. Patient had been reevaluated 4 weeks later, finding worsened aortic regurgitation (3 + /4+) with regurgitant flow directed to the anterior MV leaflet and confirming the vegetation-like formation on A2 (suspecting now a pseudoaneurysm) with worsened MV regurgitation (3 + /4+) (Figures 1 and 2).

**Figure 3:**
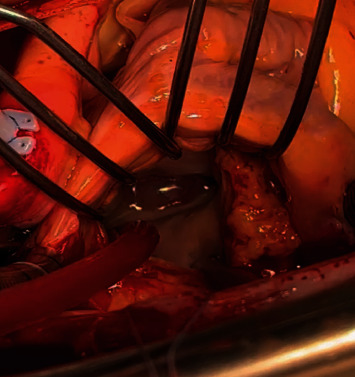
Aneurysm on the atrial aspect of the anterior MV leaflet (A2 scallop).

**Figure 4:**
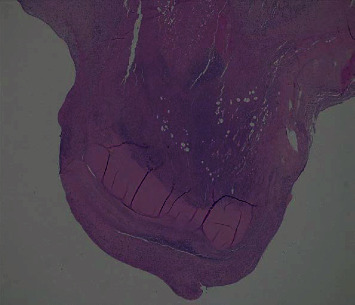
Anterior MV leaflet: slide.

After 8-week intravenous antibiotic therapy, patient was hemodynamically stable and his symptoms improved, but vegetations did not. Patient was then transferred to our center, where he underwent surgery on January. Intraoperatively, a prominent aneurysm was noted on the atrial aspect of A2, with no signs of rupture (Figure 3); apart from that, anterior and posterior MV leaflets were normal. Aortic and mitral valves have been replaced with biological prosthesis (Carpentier-Edwards PERIMOUNT Magna Ease 25 mm and Medtronic Hancock II 27 mm, respectively); left appendage closure was also performed (because of his history of AF). Intraoperative TEE showed excellent prosthesis function with no paravalvular leakage and normal EF. Microscopic evaluation of MV leaflet demonstrated acute IE, important fibrinous exudation and leukocytosis, inflammatory granulation tissue, myxoid degeneration foci, and fibrosis and adipose metaplasia (Figure 4). Postoperative time was uneventful; blood and valve cultures were negative so we stopped the antibiotic regimen. Predischarge echocardiogram showed no signs of IE. Colonoscopy was performed, demonstrating the presence of two polyps, which were later removed endoscopically.

## 3. Discussion

This is a case of *S. gallolyticus*-related native aortic valve infective endocarditis with concomitant anterior mitral valve leaflet aneurysm. Fortunately, patient underwent surgery before aneurysm rupture. We hypothesize that regurgitant infectious flow from aortic valve played a major role in the present case. Incidence of MV aneurysms is low, and echocardiographic diagnosis is made in just 0.2-0.3% of overall cases. Mostly, aneurysm is related to IE [5, 8, 11]. Mechanisms leading to aneurysm formation are well known; as described by Vilacosta et al., IE progresses to MV; an abscess develops; tissues become more and more weak, having tissue bulging; and eventually aneurysm formation [5]. Few dozens of IE-related MV aneurysms are described. Tomsic et al. did an excellent review documenting perforation in 72% of cases and embolization in 18.2% [13]. Despite that there is no specific guideline regarding surgery and MV aneurysm, we agree with Ruparelia et al.: when aneurysm is demonstrated or highly suspected, surgery should be performed as soon as possible to avoid the severe complications previously described [8]. In our case, TEE showed a vegetation-like formation on the atrial aspect of the MV, with no clear evidence nor specific sign of aneurysm. Preoperative diagnosis could underestimate the real incidence of MV aneurysms, which may mimic vegetation, tumor, prolapse, or billowing. Being IE the most common cause of MV aneurysm, we should always think about this complication. Color flow Doppler usually helps distinguish MV aneurysm from other abnormalities by demonstrating direct communication between aneurysm and left ventricle [15]. The important message we learned is to pay extreme attention on the anatomy of MV leaflets every time we face an endocarditis, always looking for something suspicious for aneurysm. Specifically, in case of AV endocarditis, we should raise suspicion for MV aneurysm. For the same reason, if we see or suspect an MV aneurysm in another clinical scenario (MV prolapse, flail, etc.), always rule out concomitant or previous IE (IE can be clinically subtle). Another debated topic is the comparison between early and late surgery. Late surgery is related with lower morbidity and mortality (preoperative antibiotic leads to reduced inflammation; then, tissues are less weak and surgery is easier). Despite that we are in accord with that point, we think it should be imperative to perform serial TEEs to rule out classical complications and unusual ones and plan surgery as soon as possible if aneurysm is present or highly suspected. Tomsic et al. concluded that urgent surgery becomes advisable if signs of HF are present [13]; our patient was stable with no pulmonary edema nor reduced EF. Arguably, delaying surgery may have increased the risk of aneurysm rupture, leading to a more complicated operation and postoperative course. For those reasons, we would suggest urgent surgery should be indicated when evidence or suspect of MV aneurysm is present. Nonetheless, indication must be discussed case by case. Intraoperative evaluation of the MV remains crucial for both diagnosis and treatment. When feasible, MV repair remains the gold standard. In case of IE, however, replacement is often more suitable [16]. Due to not favorable anatomy, we also preferred an MV replacement (bioprosthesis), achieving an excellent outcome. Finally, being *S. gallolyticus* related to colon cancer and colon polyps [17, 18], any blood culture positive for that bacterium must suggest performing a nonurgent colonoscopy. In our patient, colonoscopy was positive for intestinal polyps. There should not be any doubt on planning a colonoscopy every time we deal with *S. gallolyticus*-related IE.

## 4. Conclusion

Mitral valve aneurysm is rare but mostly associated with infective endocarditis. Transesophageal echocardiogram is crucial to determine vegetation size and mobility but also signs of concomitant abscess, perforation, and valve aneurysm. If aneurysm is present or suspected, surgery is strictly suggested due to high risk of rupture with subsequent deterioration of hemodynamics (massive acute mitral regurgitation) and systemic embolization. If blood culture is positive for *S. gallolyticus*, colonoscopy should be done to rule out colon cancer.
